# High molecular weight sodium hyaluronate improves survival of syndecan-1-deficient septic mice by inhibiting neutrophil migration

**DOI:** 10.1371/journal.pone.0250327

**Published:** 2021-04-30

**Authors:** Tuvshintugs Baljinnyam, Enkhtuya Radnaa, Casey M. Ouellette, Christina Nelson, Yosuke Niimi, Clark R. Andersen, Vsevolod Popov, Jae-Woo Lee, Donald S. Prough, Perenlei Enkhbaatar

**Affiliations:** 1 Department of Anesthesiology, University of Texas Medical Branch, Galveston, Texas, United States of America; 2 Division of Maternal-Fetal Medicine Perinatal Research, Department of Obstetrics & Gynecology, The University of Texas Medical Branch, Galveston, Texas, United States of America; 3 Department of Biostatistics, The University of Texas Medical Branch, Galveston, Texas, United States of America; 4 Department of Pathology, The University of Texas Medical Branch, Galveston, Texas, United States of America; 5 Department of Anesthesia, UCSF School of Medicine, San-Francisco, California, United States of America; Hungarian Academy of Sciences, HUNGARY

## Abstract

We investigated the role of endothelial glycocalyx and its component syndecan-1 protein in the pathophysiology of sepsis-induced vascular hyper-permeability and examined the therapeutic effects of high-molecular-weight sodium hyaluronate (HMW-SH).

**Methods:**

Sepsis was induced by cotton smoke inhalation followed by intranasal administration of *Pseudomonas aeruginosa* in female (> 6 months) Balb/c and syndecan-1 knockout mice. Survival of mice, lung capillary endothelial glycocalyx integrity, lung water content, and vascular hyper-permeability were determined with or without HMW-SH treatment in these mice. Effects of HMW-SH on endothelial permeability and neutrophil migration were tested in *in vitro* setting.

**Results:**

In septic wildtype mice, we found a severely damaged pulmonary microvascular endothelial glycocalyx and elevated levels of shed syndecan-1 in the circulation. These changes were associated with significantly increased pulmonary vascular permeability. In septic syndecan-1 knockout mice, extravascular lung water content was higher, and early death was observed. The administration of HMW-SH significantly reduced mortality and lung water content in septic syndecan-1 knockout mice, but not in septic wildtype mice. In *in vitro* setting, HMW-SH inhibited neutrophil migration and reduced cultured endothelial cell permeability increases. However, these effects were reversed by the addition of recombinant syndecan-1 ectodomain.

**Conclusions:**

HMW-SH reduced lung tissue damage and mortality in the absence of syndecan-1 protein, possibly by reducing vascular hyper-permeability and neutrophil migration. Our results further suggest that increased shed syndecan-1 protein levels are linked with the inefficiency of HMW-SH in septic wildtype mice.

## Introduction

The most common cause of death in the intensive care unit (ICU) is sepsis, a syndrome whose outcome is determined by the interaction of pathogens with the host immune system [1]. *Pseudomonas aeruginosa* (*P*. *aeruginosa*) is one of the opportunistic pathogens responsible for acute, chronic pulmonary infections [2], leading to sepsis and contributing to the mortality among ICU patients [3]. One of the detrimental complications during sepsis is vascular hyper-permeability, leading to tissue edema [1] and contributing to death. Vascular endothelial glycocalyx (EGL) is a gel-like structure that covers the lumen of the endothelium. Its length ranges between 50 and 1,000 nm [4], and it consists of transmembrane proteoglycans including glypicans and syndecans, which carry multiple heparan sulfate, chondroitin sulfate chains [5], and hyaluronan. In contrast to glycosaminoglycans (GAG), hyaluronan (hyaluronic acid) does not bind to proteoglycans, but instead interacts with cellular membrane CD44 glycoprotein; it is not sulfated and uncharged. However, it can complex with other sulfated GAGs, enabling seclusion of water and stabilizing the gel-like structure of the glycocalyx [6]. The EGL interacts with a variety of enzymes, including endothelial nitric oxide synthase, angiotensin-converting enzymes, anti-thrombin III, apolipoprotein, and chemokines. It is also involved in homeostasis, adherence of leukocyte and platelets, and transduction of shear stress signaling to the endothelium [7–10]. Importantly, EGL is recognized as a critical player of the microvascular endothelial barrier function [11].

Endothelial glycocalyx shedding can be induced by various stimuli including inflammation, thrombin, tumor necrosis factor, and lipopolysaccharides [12–16]. Increased levels of shed EGL constituent correlate with increased mortality in sepsis [17,18]. The disruption of EGL is also associated with vascular hyper-permeability to macromolecules [19] and increased recruitment of leukocytes and platelets into the site of injury [20,21]. Disruption of EGL has been found in various pathological conditions, including type I diabetes [22–24]. Inagawa et al. reported severe disruption of EGL in endotoxemia mice [25].

Syndecan-1 (Sdc-1) protein is one of the main constituents of EGL and plays an important role in endothelial integrity and barrier [7]. The Sdc-1 protein belongs to the heparan sulfate proteoglycan family and consists of a highly conserved C-terminal cytoplasmic intracellular domain, a single-pass transmembrane, and extracellular ectodomain, which carries five GAG chains. These chains display different GAG types (depending on the tissues), comprising heparan sulfate and chondroitin sulfate with varieties of length and specific structure. The Sdc-1 is mainly presented on the endothelial cell surface [26]. In response to various stimuli and disease conditions, Sdc-1 ectodomain sheds from the cell surface. It has been reported that shed Sdc-1 is increased in septic patients’ serum and that its level is significantly higher in non-survivors [27]. Also, Sdc-1 acts as a double-edged sword; in intact or full-length form, it displays anti-inflammatory properties, whereas its shed or short ectodomain plays a pro-inflammatory role. Leukocyte adherence to the endothelium is increased in Sdc-1 knockout (KO) mice [28]. In another study, the prevention of Sdc-1 shedding inhibited neutrophil transmigration and reduced inflammation in the colitis model [29].

Sodium hyaluronate (SH) is one of the components of EGL and extracellular matrix. Several receptors are known for SH, including lymphatic vessel endothelial receptor 1, SH receptor for endocytosis, toll-like receptor 4, and CD44 [30,31]. In previous studies, circulating SH was increased 4-fold in septic patients, with higher levels in non-survivors [32,33]. Depending on the molecular weight, SH can trigger either pro-inflammatory (low-molecular-weight SH [LMW-SH]) [34–37] or anti-inflammatory (high-molecular-weight SH [HMW-SH]) immune responses [34,38].

In the present study, we aimed to define the role of the EGL and Sdc-1 protein in sepsis, using a murine sepsis model, and to test the therapeutic effects of HMW-SH.

## Materials and methods

### Animal care and use

All animals were cared for following the approved protocol by the Institutional Animal Care and Use Committee (IACUC) of the University of Texas Medical Branch, and experiments were conducted in compliance with the guidelines of the National Institute of Health and the American Physiological Society for the care and use of laboratory animals. Animals were housed in an IACUC-approved facility with controlled temperature (~22°C), humidity (~50%), and a light cycle of 12 light/12 dark hours. Mice were checked every 3 hours during the study period of up to 96 hours. Animal wellbeing was determined using a customized pain and distress chart, which includes animal appearance, posture, coat, ocular discharge, level of responsiveness, and loss of body weight. When animals were found moribund, meeting the euthanasia criteria, they were humanely euthanized under anesthesia. During the 96-h survival study, 13 mice were euthanized upon reaching the criteria and 12 mice were found dead between the checks.

### Experimental design

Female Balb/c wildtype (WT) and Sdc-1 knockout mice (> 6 months) weighing 20–28 grams were used in this study. Anesthetized mice were weighed and subjected to smoke inhalation and instillation of *P*. *aeruginosa* into the nostrils or sham injury. Sham injury consisted of inhaled air instead of smoke and the same amount of PBS instilled into the nostril. Then, both WT and Sdc-1 knockout mice were randomly allocated to different groups: Sham, subjected to sham injury and treated with PBS; Untreated, subjected to smoke inhalation and instillation of *P*. *aeruginosa* and treated with vehicle (PBS); and Treatment, subjected to smoke inhalation and instillation of *P*. *aeruginosa* and treated with HMW-SH. Both WT and Sdc-1 knockout mice in the treatment group received one-time intraperitoneal injection of 20 mg/kg HMW-SH 4 hours after the induction of sepsis. Mice in sham and untreated groups received the same volume of PBS as a vehicle. Details on grouping, treatment, and number of animals are shown in Table 1. Dose and route of administration of HMW-SH were adapted from previously published work [39].

**Table 1:** Animal groups, injury, and treatment.

ANIMAL GROUPING			
MICE GENOTYPE	GROUPS	INJURY	TREATMENT
Wildtype (Balb/c)	**SHAM**	Sham injury	Vehicle (PBS)
	**UNTREATED**	Smoke+*P*.*a*	Vehicle (PBS)
	**TREATED**	Smoke+*P*.*a*	HMW-SH
Sdc-1 knockout (Balb/c)	**SHAM**	Sham injury	Vehicle (PBS)
	**UNTREATED**	Smoke+*P*.*a*	Vehicle (PBS)
	**TREATED**	Smoke+*P*.*a*	HMW-SH

Smoke: Smoke inhalation injury; P.a: Pseudomonas aeruginosa; HMW-SH: High-molecular-weight sodium hyaluronate.

After the injury (or sham injury), mice were returned to their cages and allowed to recover from anesthesia; their condition was checked every 3 hours. For the measurement of various endpoints, mice were euthanized at different time points after the injury (see S1 Illustration for the study design chart). For the survival study, mice were monitored for 96 hours at 3-hour intervals. Moribund mice were humanely euthanized upon reaching the euthanasia criteria.

### Smoke inhalation injury

Smoke inhalation was induced using a previously published method [40]. Briefly, anesthetized mice (4–5% inhaled isoflurane) were placed in the chamber and subjected to smoke inhalation. Smoke was produced by burning 40 grams of the cotton towel in a modified bee smoker and delivered (15 breaths) to the chamber via a connecting tube. Mice spontaneously inhaled four sets of smoke with a 15-second break (inhaling room air) after each set. Each smoke set lasted 1 minute. Immediately after smoke inhalation, arterial blood carboxyhemoglobin (Blood Gas System, RapidPoint 500, Siemens Healthcare, Erlagen, Germany) level was determined [40] in the representative mice to ensure the degree of injury.

### Bacterial challenge

Immediately after the smoke inhalation injury, 3.2 x 10^7^ CFUs of *P*. *aeruginosa* were introduced into the nostrils by a previously published method [40], and mice were kept in an upright position for at least 1 min to allow for sufficient entrance of bacteria into the airways. Fifteen hours after the injury, representative WT mice were euthanized to ensure bacterial migration to the lower parts of the lung. This was confirmed by a positive culture of *P*. *aeruginosa* in the lung tissue homogenate (2.5–3 x 10^4^ CFU per gram tissue). Additionally, at 18 hours after the injury, *P*. *aeruginosa* CFUs were determined in the lung tissue of sham and treatment Sdc-1 KO mice. The dose of *P*. *aeruginosa* was chosen based on a previous study comparing different doses [40].

### Determination of bacterial number in lung tissue

To determine the effect of HMW-SH on the bacterial number (CFU), untreated vs. treatment groups of Sdc-1 KO mice were euthanized at 18 hours after the induction of sepsis. Subsequently, the lung tissue was meshed through a cell strainer (70 um) in 1 ml PBS, and the obtained homogenate was diluted (according to pre-determined dilution factor) and spread on the trypticase soy agar plate (#212305, BD) (6 mice per group). Randomly selected colonies were further confirmed by PCR using specific primers designed for *P*. *aeruginosa* (forward: GGGGGATCTTCGGACCTCA and reverse: TCCTTAGAGTGCCCACCCG) [41] (please see S1D Fig for detailed information in S1 Raw images).

### Visualization of vascular EGL

Lung capillary EGL was visualized in WT mice by a method previously described by Van den Berg *et al*. [42]. For the comparison sham vs. untreated, mice (analysis at 16 and 24 hours after the induction of sepsis) were fully anesthetized and perfused with cardioplegic solution and primary fixative solutions, followed by perfusion with primary fixative solution containing 0.05% Alcian blue. Then, the lung was dissected out and fixed in fixative solution (4% PFA and 1% GA) and post fixed (1% osmium tetroxide and 1% lanthanum nitrate), followed by 1% aqueous uranyl acetate. After being dehydrated in alcohol and propylene oxide, it was embedded in Poly/Bed 812. Ultra-thin sections of capillaries were cut on a Leica EM UC7 ultramicrotome (Polysciences, Warrington, PA, USA), and cross-sections on copper grids were visualized with a Philips (FEI) CM-100 electron microscope at 60kV and quantified with the ImageJ software. The EGL of the lung capillary was semi-quantified by a masked investigator. The total area of the EGL was determined in a single mouse for each measured timepoint, using the ImageJ software, and the numbers were normalized to the perimeter of the capillary (total 500 micrometers of capillaries per timepoint).

### Vascular permeability assay

To evaluate vascular leakage in WT mice, 0.5% Evans blue salt solution was intravenously injected 30 minutes before euthanasia at 0, 12, 15, 18, and 21 hours after the induction of sepsis. Lung tissues were collected after the perfusion of pulmonary vasculature with PBS (5 ml) and meshed through a 70-um filter. The resulting filtrate was incubated in formaldehyde at 55°C for 24–48 hours. After a spin at 2,000 rpm for 5 minutes, the cleared aqueous solution was used for determination of Evans blue salt concentration via spectrophotometry at 620 nm (10 mice per timepoint).

### Measurement of shed Sdc-1

Serum was prepared from healthy and septic WT mice euthanized at 12, 15, 18, 21, and 24 hours after the induction of sepsis (4 mice per timepoint). After the separation of serum proteins in tris-glycine gel, proteins were transferred to the polyvinylidene difluoride (PVDF) membrane and incubated with anti-syndecan-1 antibody [43,44] and HRP-conjugated goat-IgG. The HRP signal was detected using ECL substrate, exposed to the X-ray film, and developed with a Kodak X-OMAT 2000A processor. The original immunoblotting images are presented in S1A-S1C Fig in S1 Raw images.

### Lung water content

Eighteen hours after the induction of sepsis, untreated groups of WT vs. Sdc-1 KO mice were euthanized (10 mice per group). The lungs were removed and weighed to assess the wet weight (WW), followed by drying for 24 hours at 110°C to determine the dry weight (DW). The lung water content was calculated using the following formula: W/D weight ratio = WW/DW.

### Isolation of neutrophils

To obtain the neutrophils, mice were injected intraperitoneally with 0.4% glycogen by a previously published method [45,46]. Six hours after the injection, peritoneum was lavaged with 5 ml of neutrophil isolation medium, and the cell suspension was passed through a cell strainer (70 um). The resulting cell suspension was used for further separation of neutrophils by centrifugation in 63% Percoll solution. Contaminating red blood cells were lysed and washed away. After confirmation of the cell viability by trypan blue staining, the purity of the neutrophils was determined by Wright’s staining and DAPI staining. Viability and neutrophil purity were 98% and 92–96%, respectively. Viability of the cells were not affected by the addition of either HMW-SH or recombinant Sdc-1 ectodomain (S1 Table in S1 Raw images).

### *In vitro* migration assay

Neutrophil migration was evaluated by a previously described method [45] with slight modifications, using Transwell (3.0-uM pore diameter) inserts (Corning, Life Science). Neutrophils were added to the upper chambers with or without HMW-SH (2 ug/ml), or recombinant Sdc-1 (200 ng/ml), or both. Neutrophil migration was induced by the addition of casein to the bottom chambers. The chemotaxis result was evaluated by counting cells migrated across the filter and presented as percentage of input. Experiments were repeated independently 4 times.

### *In vitro* endothelial permeability assay

Endothelial permeability was examined as previously described [47], with modifications. The HMVEC-L cells were seeded at confluent as a monolayer and allowed to adhere to the filter of the collagen pre-coated Transwell inserts (Corning, Life Science) overnight. Four hours prior to the experiments, endothelial cells were activated by TNF-alpha (20 U/ml). Activation of endothelial cells was confirmed by the presence of stress fiber. Freshly isolated neutrophils were added to the upper chamber in combination with HMW-SH (2 ug/ml) and/or recombinant Sdc-1 (200 ng/ml). At 1 hour after co-incubation, the fraction of medium transferred to the opaque 96-well plate and the amount of FITC-Dextran (40 kDa) diffused to the lower chamber were measured using a spectrofluorometer. Breifly, after collection of medium from the lower chambers (including negative control well, where HMVEC-L cells were cultured and treated same way, except no FITC-Dextran and neutrophils were added), mediums were diluted and transferred to the opaque 96 well plate. Fluorescence level was determined and recorded. Obtained numbers were multiplied by the dilution factor to get absolute fluorescence level. Then value for negative control was subtracted from all sample values to remove any autofluorescence, if there was any. Finally, all values were normalized by the corresponding value for “HMVEC-L” (no neutrophils) well to get relative permeability to undisturbed endothelial permeability. Result are shown in arbitrary unit (AU). The experiments were repeated independently 3 times.

### Histopathological analysis

Four micrometer sections were cut from paraffin blocks of formalin-fixed lung tissues and stained with hematoxylin and eosin. The scores of the tissue damage in the lungs were evaluated as described previously [48,49]. Briefly, captured images were coded and transferred to the investigator to be blindly scored as 1 to 5. After scoring, the obtained data were returned to a second investigator to be de-coded and analyzed. Images were captured with Keyence (BZ-X810, Itasca Il, USA) (4 mice per group).

### Neutrophil count

To determine the number of circulating neutrophils, arterial blood was collected at 18 hours after the induction of sepsis and analyzed using a complete blood cell count analyzer (ADVIA120 hematology system, Malvern, PA, USA) (n = 10 per group).

### Reagents and antibodies

High-molecular-weight sodium hyaluronate was purchased (#HA1M-1, Lifecore). Human lung microvascular endothelial cells (HMVEC-L) (#CC2527, Lonza) and endothelial cell growth medium with supplements (#MD-0010 and #MD-00106, iXCells Biotechnology) were purchased. Recombinant Mouse Syndecan-1 (amino acids 18–252 as cleaved extracellular part) (#P18828, RnD), anti-mouse Sdc-1 antibody (#60035, Stemcell) 281–2 (reacts with shed Sdc-1 form only [43]) [44], dextran-FITC 40kDa (#FD40, Sigma Millipore), goat anti-rat IgG HnL HRP (#ab97075, Abcam), and black, opaque 96-well plates (#3915, Corning) were purchased. *Pseudomonas aeruginosa* (Schroeter) was purchased (#27317, ATCC), cultured, and handled in accordance with the regulation of BSL2.

### Statistical analysis

Statistical analysis was performed using the GraphPad Prism 8 software. Significance was determined using either one-way or two-way *analysis of variance (ANOVA)*, *when appropriate*, *followed by Bonferroni post-hoc test multiple comparisons*. The correlation between EGL integrity and hyper permeability was calculated via linear regression; *p* < 0.05 was considered statistically significant. Survival was analyzed by the Kaplan-Meier survival curve method for 94 hours, and death incidence at 24 hours was calculated using a two-sided Fisher’s exact test.

## Results

### Disruption of vascular EGL in WT mice

**Fig 1:**
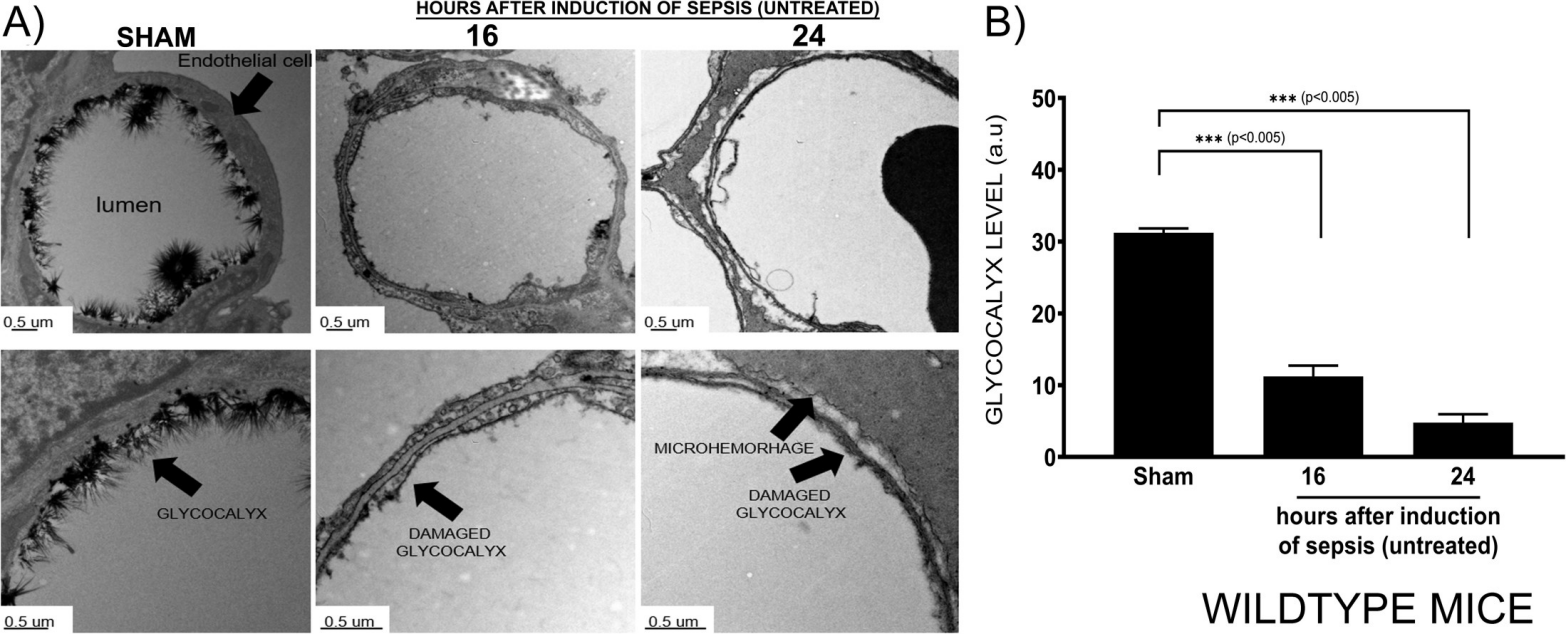
Disruption of pulmonary vascular EGL in sepsis induced by double-hit injury. In healthy and septic WT mice, at the indicated time points, pulmonary microvascular endothelial glycocalyx (A) was visualized by Alcian blue staining and transmission electron microscopy. Significant decreases in the pulmonary microvascular endothelial glycocalyx (semi-quantitative score) were observed at 16 and 24 hours after the induction of sepsis (500 micrometers of capillaries per measured timepoints) (B). Data are expressed as mean ± SEM (*p < 0.05, **p < 0.01).

**Fig 2:**
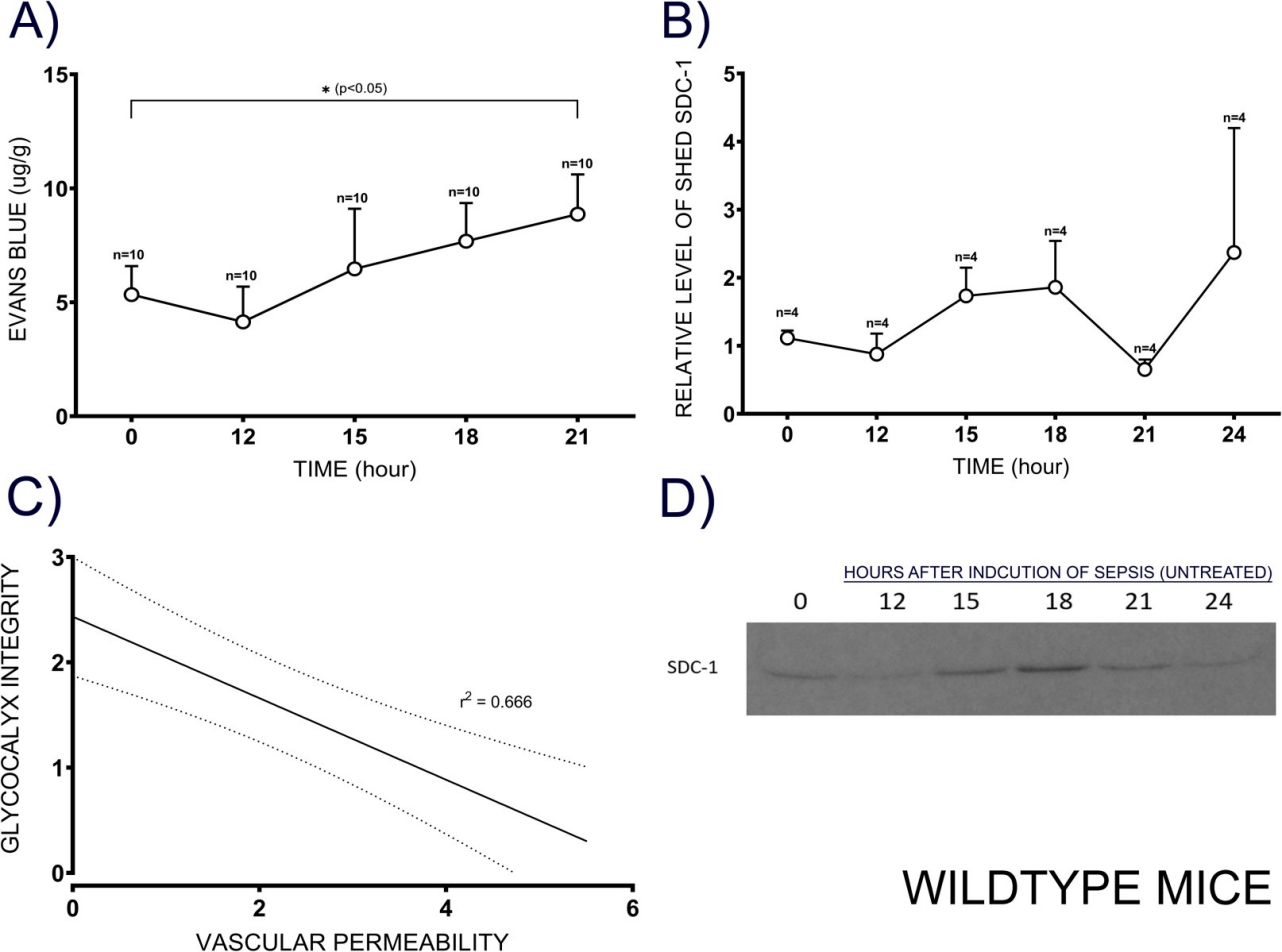
Association between vascular hyper-permeability and integrity of EGL. To evaluate vascular hyper-permeability (A), Evans blue dye was intravenously injected into WT mice (10 animals per time point) 30 min before euthanasia. The free dye was washed out, and the Evans blue concentration in the lung homogenate was determined at 620 nm. Circulating shed Sdc-1 was measured in the serum collected (4 mice per time point) at indicated time points after the induction of sepsis by immunoblotting (B, D). A moderate negative association was found between glycocalyx integrity and vascular hyper-permeability (C), and the non-continuous line indicates confidence interval (95%). Data are expressed as mean ± SEM (*p < 0.05).

The EGL was intact in the healthy lung, whilst severe disruption of EGL was observed at 16 (*p <* 0.005) and 24 hours (*p <* 0.005) after the induction of sepsis (Fig 1A and 1B) in WT mice. Shed Sdc-1 protein levels were gradually increased in the serum, reaching the highest level at 18 and 24 hours in untreated septic WT mice (*p =* 0.26), whereas the levels were unchanged in Sham WT mice (Fig 2B and 2D).

### Pulmonary microvascular hyper-permeability in WT mice

Pulmonary microvascular hyper-permeability in sham and untreated WT mice was determined using Evans blue injection method. It was gradually increased in untreated septic WT mice, reaching a significant difference (*p =* 0.04) at 21 hours after the induction of sepsis compared to sham (Fig 2A). A moderate correlation (r^2^ = 0.67, slope -3.8) was found between the integrity of EGL and vascular hyper-permeability; with every 1-unit decrease in glycocalyx integrity, hyper-permeability was increased by 3.8 ± 1.2 units (*p <* 0.05) (Fig 2C).

### Early death of Sdc-1 knockout mice

**Fig 3:**
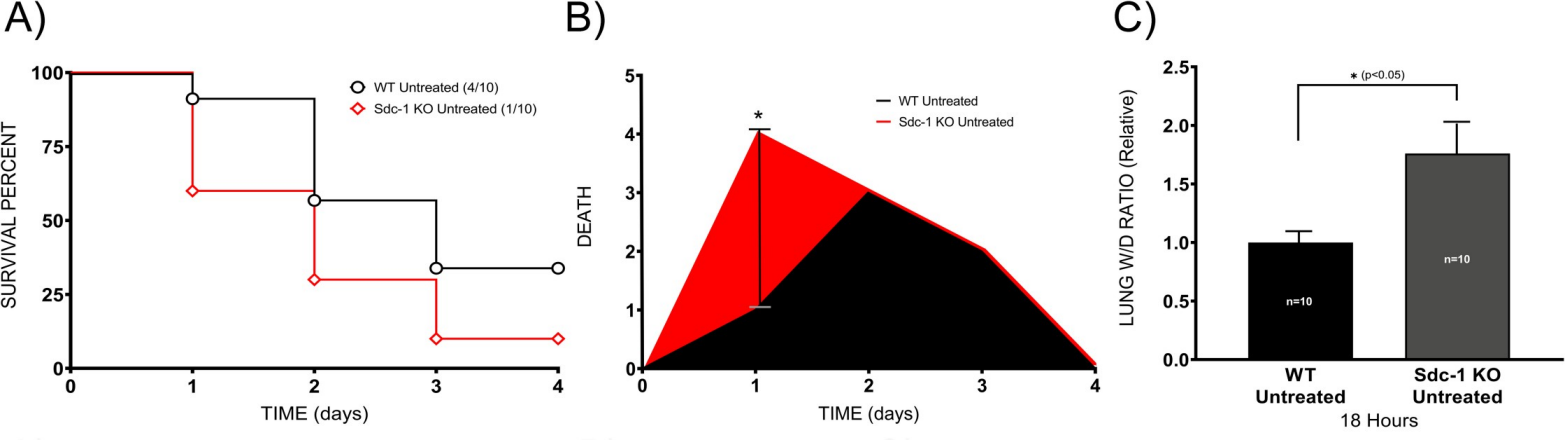
Sdc-1 KO mice are susceptible to sepsis. WT and Sdc-1 KO mice received a double hit injury of smoke and bacteria, and their survival was monitored for 96 hours (10 mice per group) (A). Death incidence was determined in relation to time, and results are shown in the area under the curve (B). Lung water content was determined at 18 hours by measuring the lung W/D weight ratio (10 mice per group) (C). Data are expressed as mean ± SEM (*p < 0.05).

To understand the role of the Sdc-1 protein in sepsis, we compared the survival of WT and Sdc-1 KO mice for 96 hours after the induction of sepsis. At the end of the experiment, the percentage of survival was 10 vs. 40% for Sdc-1 KO and WT mice, respectively (Fig 3A) (*p =* 0.08). Early death was observed in Sdc-1 KO mice; by 24 hours after the induction of sepsis, the incidence of death was significantly higher (*p =* 0.024) in Sdc-1 KO mice (40%) than in WT mice (10%) (Fig 3B).

Subsequently, we evaluated the lung water content (wet-to-dry weight [W/D] ratio) of the lungs from Sdc-1 KO and WT mice. We chose 18 hours to collect lungs despite the highest increases of shed Sdc-1 were found at 18 and 24 hours because of high mortality rate at the 24 hours. The lung W/D dry weight ratio was significantly elevated (*p <* 0.05) in the untreated septic Sdc-1 KO compared to untreated septic WT mice (Fig 3C).

### Sodium hyaluronate improved survival of septic Sdc-1 knockout mice

**Fig 4:**
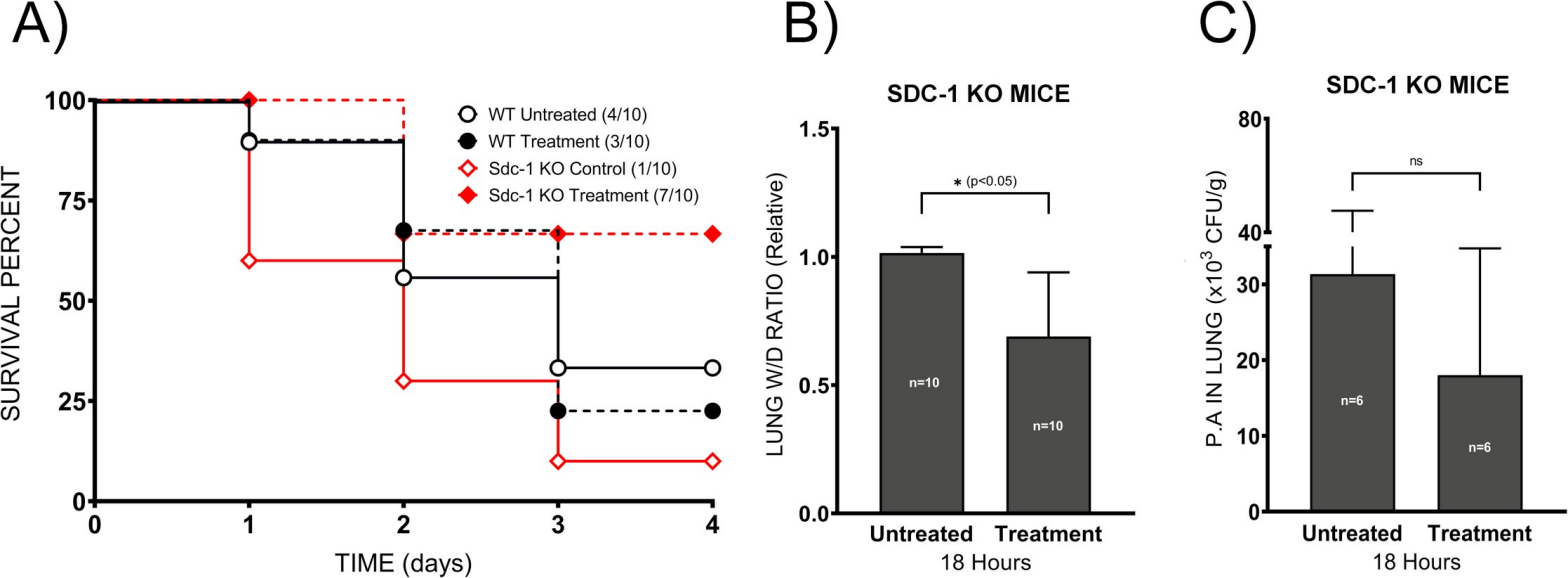
Administration of HMW-SH improves survival of septic Sdc-1 KO mice. The therapeutic effect of HMW-SH was tested in WT and Sdc-1 KO mice by intraperitoneal injection of HMW-SH at 4 hours after the induction of sepsis, and their survival was monitored for 96 hours (10 mice per group) (A). To compare the lung water contents of the septic Sdc-1 KO mice that received vehicle or HMW-SH administration, lung wet/dry weight ratio was calculated (10 mice per group) (B). Bacterial number in the lung tissue was determined 18 hours after the induction of sepsis in Sdc-1 KO mice with or without HMW-SH administration (6 mice per group) (C). Data are expressed as mean ± SD (*p < 0.05).

Administration of HMW-SH exhibited no effect on WT mouse mortality. However, survival of Sdc-1 KO mice was improved (*p <* 0.05) by 60% with the treatment (Fig 4A). Further, we compared the lung water content in the lungs of septic Sdc-1 KO mice with or without HMW-SH treatment. At 18 hours after the induction of sepsis, the lung water content was significantly reduced (*p <* 0.05) in the HMW-SH treatment group (Fig 4B).

### Bacterial clearance

Next, we determined whether the reduction of lung water content was related to improved bacterial clearance by HMW-SH in the Sdc-1 KO mice. We found that HMW-SH administration had no effect (*p =* 0.62) on bacterial clearance (Fig 4C).

### Lung tissue histology

**Fig 5:**
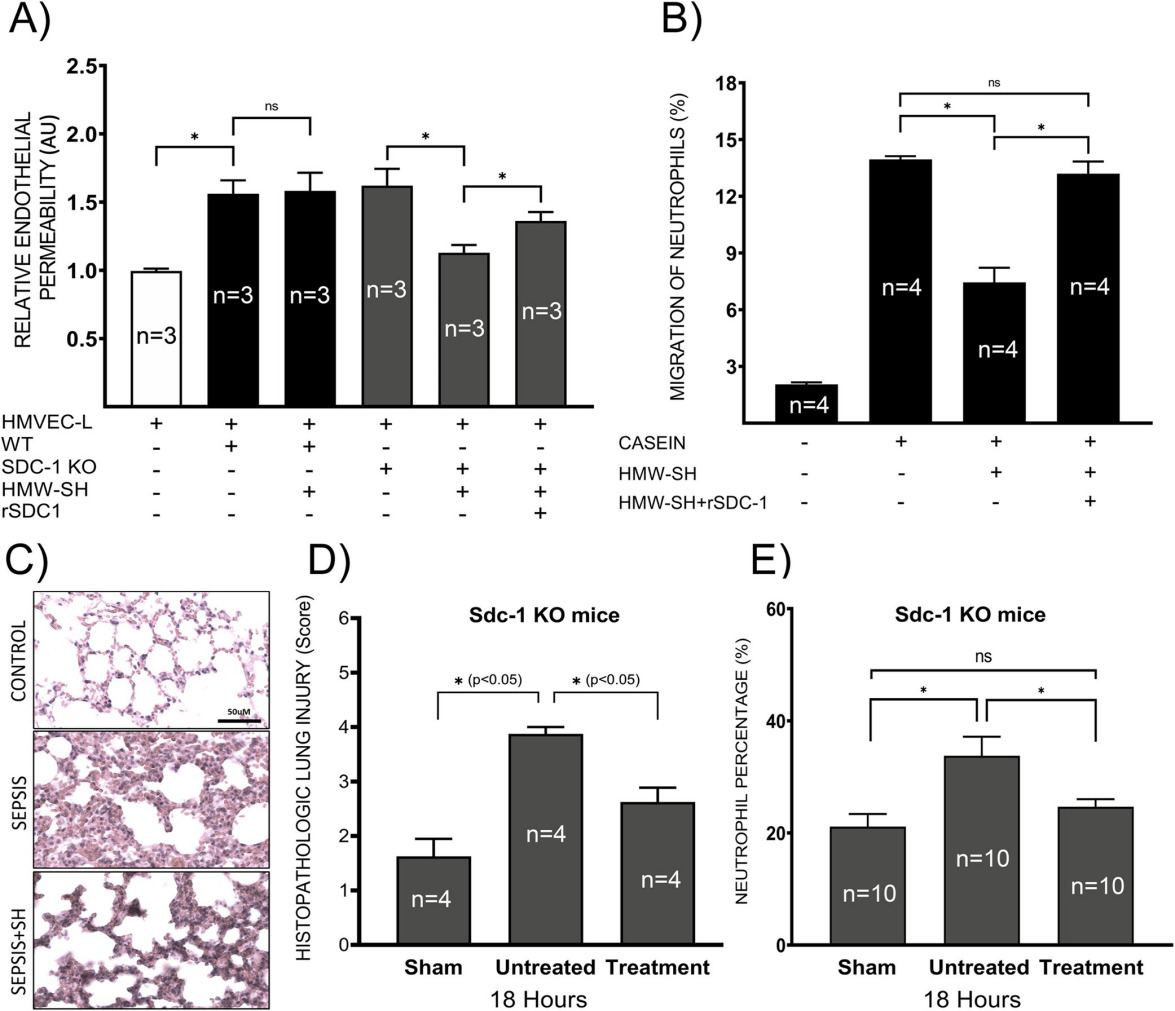
Shed Sdc-1 protein interferes with the effect of HMW-SH. The effect of HMW-SH on endothelial permeability was examined *in vitro* using neutrophils isolated from WT and Sdc-1 KO mice (experiments repeated 3 times independently) (A). Co-culture of neutrophils with the endothelial cells significantly increased endothelial permeability, regardless of the genotype (isolated from WT and Sdc-1 KO mice). The addition of HMW-SH reduced the permeability induced by neutrophils derived from Sdc-1 KO mice, which was reversed by the addition of recombinant Sdc-1 protein. However, HMW-SH failed to reduce endothelial permeability induced by neutrophils derived from WT mice. Neutrophil migration was evaluated *in vitro*, results are presented as the percentage of input (experiments repeated 4 times independently) (B). Lung tissue damage was evaluated by the histopathological scoring of lung tissue based on H&E-stained sections (4 mice per group), representative images of lung Sdc-1 KO mice are shown (C), and quantified lung injury scores for indicated groups are shown (D). Finally, the circulating neutrophil percentage was compared in Sdc-1 KO mice with or without HMW-SH treatment (10 mice per group) (E). Data are expressed as mean ± SEM (*p < 0.05).

Further, we examined lung tissue damage in septic Sdc-1 KO mice treated with or without HMW-SH. The lung tissue injury score was significantly lower (*p <* 0.05) in HMW-SH-treated mice compared to untreated mice (Fig 5C and 5D).

### Effects of neutrophils on cultured endothelial cell permeability *in vitro*

Neutrophils isolated from WT and Sdc-1 KO mice were co-incubated with cultured HMVEC-L cells in the media containing dextran-conjugated FITC. Endothelial cells were activated by TNF-alpha. Next, HMW-SH and/or recombinant Sdc-1 protein were added to the upper chamber, and 1 hour after incubation, the concentration of FITC was measured in the lower chamber medium. We found that the presence of neutrophils significantly increased (*p <* 0.005) endothelial permeability at a similar extent, regardless of the origin of neutrophils (Fig 5A). Interestingly, increased endothelial permeability, mediated by the Sdc-1 KO neutrophil, was attenuated by the addition of HMW-SH (*p <* 0.005). In contrast, HMW-SH did not affect permeability changes induced by WT mouse neutrophils (Fig 5A) (*p =* 0.89). The reduction of endothelial permeability induced by Sdc-1 KO mouse neutrophils was reversed by the addition of recombinant Sdc-1 ectodomain (Fig 5A) (*p <* 0.05).

### Neutrophil migration assay

Since altered transmigration of neutrophil could affect endothelial permeability, we examined the effect of HMW-SH on the neutrophil transmigration *in vitro*, as previously described [50]. Significantly reduced transmigration of neutrophils through filter pores by HMW-SH treatment (*p <* 0.005) was reversed by the addition of recombinant Sdc-1 to the medium (Fig 5B) (*p <* 0.005). This result was supported by the data obtained from Sdc-1 KO mice (Fig 5E), where we found a significantly lower percentage of neutrophils in the circulation of HMW-SH-treated septic Sdc-1 KO mice (20%) compared to untreated septic Sdc-1 KO mice (30%) (*p <* 0.01).

## Discussion

In the present study, we explored the roles of vascular EGL and Sdc-1 protein in the pathophysiology of sepsis and tested the therapeutic effects of HMW-SH, using the setting of the double-hit injury murine model (Sdc-1 KO and WT mice) of sepsis [40]. We found elevated levels of shed Sdc-1 protein in the circulation and severe damage of the lung capillary EGL in septic WT mice. These changes were associated with increased vascular permeability.

Our data confirmed that disruption of the EGL makes pulmonary vasculature more prone to leak in sepsis, and the absence of Sdc-1 protein results in the early death of septic Sdc-1 KO mice, with significantly increased lung water content compared to septic WT mice. Administration of HMW-SH exhibited no effect on the mortality of septic WT mice, although it improved the survival of Sdc-1 KO mice. The improved survival in Sdc-1 KO mice was associated with reduced vascular hyper-permeability and lung tissue damage score, as well as reduced neutrophil migration. While other mechanisms, including mitochondrial dysfunction, circulatory shock, and hypoglycemia, may have contributed to death, our data indicate a critical role of EGL in the survival of these septic mice.

Pulmonary edema is a detrimental complication in septic patients, contributing to the severity of multiorgan dysfunctions and death. The EGL has been reported as an important factor in vascular endothelial barrier integrity and regulating vascular permeability [7]. Its disruption has previously been documented in the myocardial and pulmonary capillaries of septic mice [4,42,51]. However, the role of EGL in the pathophysiology of sepsis remains not completely understood. Our results demonstrate that pulmonary vascular EGL is disrupted as early as 16 hours after sepsis induction, which was associated with lung tissue microhemorrhage (at 24 hours after the induction of sepsis). We report that disruption of vascular EGL, evidenced by its ultrastructural damage, was associated with vascular hyper-permeability and increased mortality in our double-hit injury sepsis model.

Since Sdc-1 is one of the major constituents of EGL, we compared the survival of septic Sdc-1 KO mice vs. septic WT mice. Interestingly, Sdc-1 KO mice exhibited a significantly increased incidence of early death (at 24 hours) compared to WT mice. This survival study result is not consistent with the result obtained from the skin thermal injury-induced sepsis model, where death was delayed in septic Sdc-1 KO mice [52]. Although the reason for this discrepancy remains unknown, it could be explained by the study model differences (pulmonary vs. non-pulmonary sepsis), including the degree of severity of microvascular hyper-permeability in the lungs. It may also be related to the fact that shed Sdc-1 protein can act as a pro-inflammatory agent, the impact of which may differ depending on the disease process. Nevertheless, the significantly increased lung extravascular water content in septic Sdc-1 KO mice compared to WT mice suggests that disruption of the EGL plays a critical role in pulmonary microvascular hyper-permeability and increased mortality in septic mice. Although the exact reason remains unknown, we speculate that differences in septic WT and Sdc-1 KO mice microvascular permeability are not mediated by the severity of bacterial infection. In a previous study, *P*. *aeruginosa* entrance to circulation was not dependent on the presence of Sdc-1 on the cell surface [53]. It has also been reported that bacterial infection and propagation were not affected by Sdc-1 [52], supporting our notion.

Neutrophil is the first defense agent against bacterial infection; however, excessive infiltration of neutrophils could damage the target organ. Particularly, an elevated percentage of neutrophils increases the probability of adherence to the vascular endothelium, leading to vascular endothelial integrity barrier disruption and hyper-permeability. Based on a previous study, exogenous SH inhibits the adhesion of leukocyte and platelets in conditions of both turbulent flow and constant shear rate [54]. The authors noted that this effect was not due to a change of solution viscosity [54] but rather related to SH, which is an important constituent of EGL.

In the present study, intraperitoneal administration of HMW-SH significantly reduced lung water content and improved the survival of septic Sdc-1 KO mice, suggesting that HMW-SH restored endothelial barrier function and reduced vascular hyper-permeability. This notion is supported by the report of Chappell et al. that protection of EGL reduced leukocyte adhesion to the endothelium and reduced tissue edema after ischemia/reperfusion [55]. The fact that the bacterial number in the lung tissue of septic Sdc-1 KO was comparable regardless of the HMW-SH treatment indicates that salutary effects of HMW-SH were not related to the bacterial clearance rate.

Interestingly, the administration of HMW-SH failed to improve the survival of the septic WT mice, although it significantly improved the survival of septic Sdc-1 KO mice. Although the exact mechanism remains unclear, these results suggest possible harmful effects of circulating shed Sdc-1 in sepsis of pulmonary etiology, which is consistent with a previous report that administration of recombinant Sdc-1 ectodomain resulted in increased susceptibility of newborn Sdc-1 KO mice to infection with *P*. *aeruginosa* [53]. Previously, it has been suggested that Sdc-1 could trigger both pro-inflammatory and anti-inflammatory signaling, depending on its status: either shed (short) or intact (full length), respectively [28,29]. The results of our and a previous study [53] suggest that shed Sdc-1 protein in WT mice attenuated the effects of HMW-SH.

An interesting finding, in the present study, was related to fact that permeability increases in cultured endothelial cells were comparable when co-incubated with neutrophils isolated from either WT or Sdc-1 KO mice. However, treatment with HMW-SH reduced the permeability of these endothelial cells only in a setting where they were challenged with neutrophils isolated from Sdc-1 KO mice. To note, the salutary effects of HMW-SH treatment were reversed by the addition of recombinant Sdc-1 ectodomain. Although the exact mechanism is unknown, we speculate that Sdc-1 protein on neutrophils (or in its shed form) of WT mice was attributable to neutrophil-endothelial interaction. The fact that the addition of the Sdc-1 protein halted the efficacy of HMW-SH indicates an important role of shed Sdc-1 in neutrophil-endothelial interaction.

In the present study, we report that the migration of neutrophils in response to casein, through filter pores, was significantly inhibited with HMW-SH co-incubation. The observed inhibition of neutrophils by HMW-SH was reversed with the addition of recombinant Sdc-1 ectodomain. These results indicate that HMW-SH inhibits transmigration of neutrophils; however, the presence of Sdc-1 protein interferes with the HMW-SH effect. This mechanism is also in agreement with the results obtained by Balazs *et al*., demonstrating inhibition of leukocyte migration by the HMW-SH from the capillary tubes [56]. To note, HMW-SH did not affect bacterial clearance. These results suggest that the transmigration of neutrophils was independent on the presence of bacteria. Findings that overexpression of mutant Sdc-1 (cleavage resistant form) in intestinal epithelial cells resulted in reduced inflammation and impaired transmigration of neutrophil [29] may support our results.

This study has a few limitations: 1) the mechanism(s) of Sdc-1 and HMW-SH actions was/were not elucidated; 2) although the migration of neutrophils was determined, the adherence of neutrophils to the endothelial cells were not studied; 3) the differences in neutrophil functions isolated from WT vs. Sdc-1 KO remain unknown; 4) although we have compared the lung wet/dry weight ratio in wildtype and Sdc-1 KO mice following the injury, we did not compare it in these mice after the treatment; 5) food consumption was not quantified (no gavage feeding); 6) the possibility that shed Sdc-1 contributed to the cleavage or degradation of the HMW-SH into LMW-SH was not tested; and 7) the inability to determine the exact cause of death. Finally, we recognize that the interrelationship between the data we present is not causative; rather, they represent co-association.

Nevertheless, our results indicate that HWM-SH reduces the mortality of septic Sdc-1 mice, possibly by inhibiting neutrophil migration and reducing pulmonary microvascular hyper-permeability. Our findings also indicate that elevated levels of shed Sdc-1 protein interfere with the effects of HMW-SH.

In summary, our data demonstrate that disruption of EGL makes vasculature more prone to leak in septic conditions. Further, administration of HMW-SH reduces lung tissue damage and mortality in the absence of Sdc-1 protein, possibly by inhibiting neutrophil migration and reducing pulmonary vascular hyper-permeability. Hence, HMW-SH should be considered as a potential therapeutic agent for the management of vascular leak and tissue edema during sepsis.

## Supporting information

S1 Checklist *PLOS ONE* humane endpoints checklist. (DOCX)

S1 Raw images (PDF)
