# Supplementary material for: High molecular weight sodium hyaluronate improves survival of syndecan-1-deficient septic mice by inhibiting neutrophil migration
Source: PLoS One. 2021 Apr 30;16(4):e0250327. doi: 10.1371/journal.pone.0250327 (PMC8087021; doi:10.1371/journal.pone.0250327)
Supplement: S1 Raw images — (PDF) [file pone.0250327.s002.pdf]

Supporting information.

Supplementary Fig 1(A-C). Detection of shed syndecan-1 protein in the serum by western blotting. Serum was prepared from blood collected from healthy (Sham), and septic mouse (at 12, 15, 18, 21, and 24 hours). After separation in tris/glycine gel, proteins were transferred to the PVDF membrane, and immunoblotted for syndecan-1. HRP signals were detected using ECL substrate and exposed to the X-ray film and developed with "Kodak X-OMAT 2000A processor". Then photograph of X-ray film was captured using "Canon EOS rebel" camera and transferred to the computer. (Figure 2D was generated from S1A figure).

Supplementary Fig 1D. Confirmation of Pseudomonas aeruginosa by PCR. To confirm if grown bacteria is *Pseudomonas aeruginosa* or not, randomly selected colonies (positive culture from lung tissue homogenate) were mini-cultured, genomic DNA was isolated. Polymerase chain reaction was performed with isolated DNA samples using set of primer specific (Spilker T, Coenye T, Vandamme P, LiPuma JJ. PCR-based assay for differentiation of pseudomonas aeruginosa from other pseudomonas species recovered from the cystic fibrosis patients. Journal of Clinical Microbiology, (2004) 5(42): 2074-2079) for *Pseudomonas aeruginosa*.

Primer sequence:

Forward: GGGGGATCTTCGGACCTCA

Reverse: TCCTTAGAGTGCCCACCCG

Image was captured using GelDoc-It TS3 Imager (Ultra-Violet Products Ltd. Trinity Hall Farm Estate Nuffield road, Cambridge CB4 1TG UK).

Legends to supplementary figures

S1 Fig.A. Shed syndecan-1 in the serum. Serum proteins separated on PAGE (16%) and immunoblotted for syndecan-1. Lane 1: MW Marker 2: (+) positive control, 3: Sham, 4: 12 hours, 5: 15 hours, 6: 18 hours, 7: 21 hours, and 8: 24 hours. Serum collection timepoints are indicated as hours.

S1 Fig.B Shed syndecan-1 in the serum. Serum proteins separated on PAGE (16%) and immunoblotted for syndecan-1. Lane 1: MW Marker, 2: Sham, 3: 12 hours, 4: 15 hours, 5: 18 hours, 6: 21 hours, and 7: 24 hours. Serum collection timepoints are indicated as hours.

S1 Fig.C Shed syndecan-1 in the serum. Serum proteins separated on PAGE (16%) and immunoblotted for syndecan-1. Lane 1: MW Marker 2: Sham, 3: 12 hours, 4: 15 hours, 5: 18 hours, 6: 21 hours, and 7: 24 hours. Serum collection timepoints are indicated as hours.

S1 Fig.D Confirmation of *Pseudomonas aeruginosa* by PCR.

PCR products run on 0.8% agarose gel. Expected PCR product size 956bp. 1: 1kb Plus marker, 2: negative control (PCR reagents excluding sample), 3: clone #8, 4: clone #14, 5: clone #48, and 6: clone #56.

S1 Figure.

A)

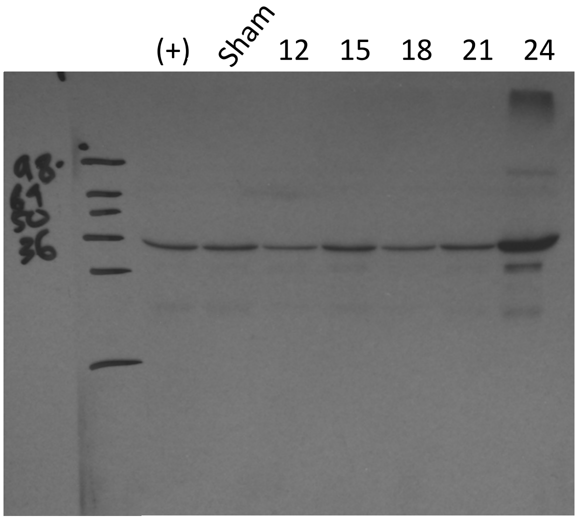

B)

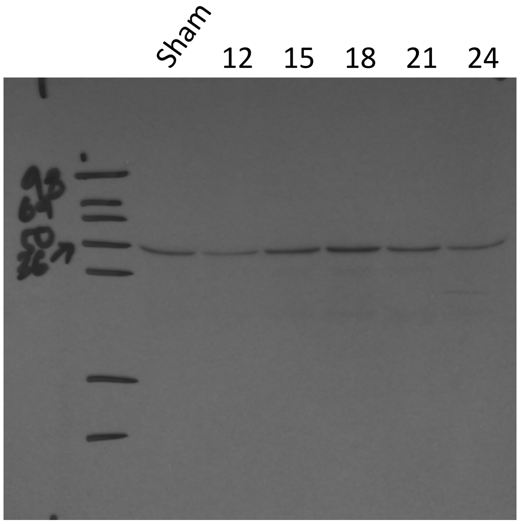

C)

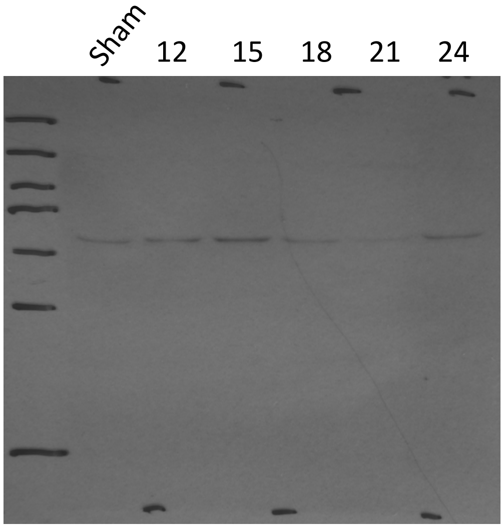

D)

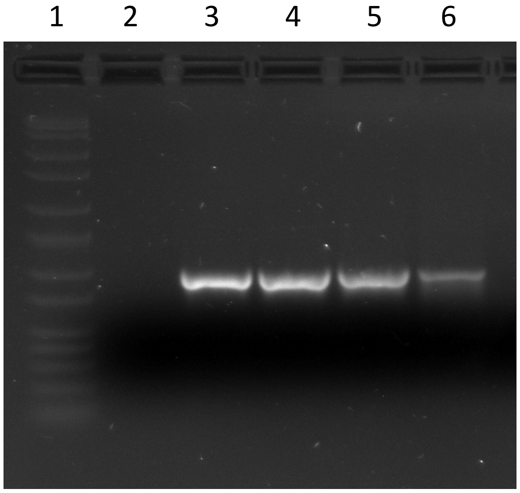

S1 Illustration. Study design.

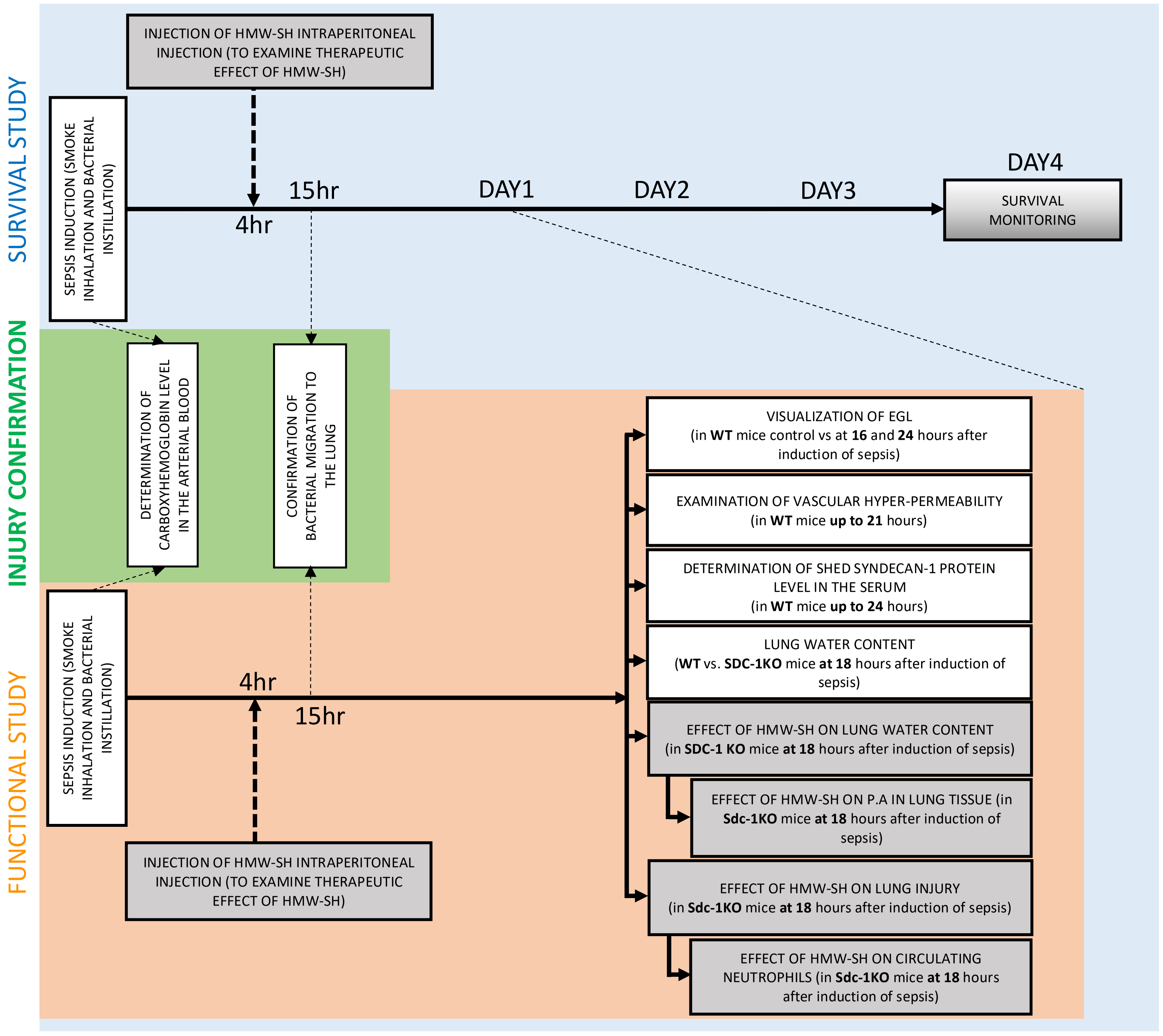

S1 Table. Purity and viability of neutrophils.

ISOLATION	COUNT (REPEATS)	NEUTROPHIL (#)	TOTAL CELL (#)	PURITY (%)	VIABILITY (%)
1	1	84	88	95.4	97
	2	78	84	92.8	
	3	86	90	95.5	
	4	90	97	92.7	
2	1	77	82	93.9	98
	2	72	76	94.7	
	3	84	87	96.5	
3	1	91	96	94.7	98
	2	92	96	95.8	
	3	88	93	94.6	
	**Average**			**94.7**	**97.6**
